# A low-loss molybdenum plasmonic waveguide: perfect single-crystal preparation and subwavelength grating optimization

**DOI:** 10.1515/nanoph-2023-0480

**Published:** 2023-10-25

**Authors:** Tao Cui, Yan Shen, Ao Cheng, Runze Zhan, Zebo Zheng, Bo Tian, Jia Shi, Yanlin Ke, Lei Shao, Huanjun Chen, Shaozhi Deng

**Affiliations:** State Key Laboratory of Optoelectronic Materials and Technologies, Guangdong Province Key Laboratory of Display Material and Technology, School of Electronics and Information Technology, Sun Yat-Sen University, Guangzhou 510275, China

**Keywords:** plasmonic waveguide, near-infrared, low-loss propagation, molybdenum single-crystal, subwavelength grating

## Abstract

Plasmonic waveguides have attracted tremendous interest due to efficiently confining photons on the subwavelength spatial scale to be beating the propagation diffraction limit. Transition metal molybdenum (Mo) exhibits outstanding properties in light trapping and electromagnetic field confining, making it potentially valuable in 1.55 μm plasmonic waveguide applications. However, the reliable fabrication of high-quality Mo plasmonic waveguides is a significant challenge. A real-space micro-imaging study of the surface plasmon on Mo structures is still absent. In this study, we successfully prepared a single-crystalline Mo microrod waveguide structure and fabricated subwavelength gratings on it. The diffraction gratings were designed, optimized, and etched to excite the surface plasmon polariton behaviour of Mo for the first time. The grating-optimized Mo microrod single-crystal reveals highly efficient waveguide performance around near-infrared spectroscopy, exhibiting a long propagation length of 32 μm and a low transmission loss of 0.067 dB μm^−1^. The results provide an alternative to advanced materials research and optical device applications of plasmonic waveguide systems.

## 1 Introduction

Plasmon has been frequently introduced to deal with the propagation diffraction limit in the last few years due to its unique advantages in high-speed propagation and tunability [1–3]. Plasmonic waveguide materials could therefore become the key components to the future high-speed optical transmission platforms in chip-to-chip interconnect over short-distance local wires and long-distance globe wires [4, 5]. Surface plasmon polariton (SPP), coupling surface oscillations of charge to electromagnetic waves, has exhibited specific capability in confining and manipulating light at the subwavelength scale [6]. This capability significantly enhances the transmission properties of optical waveguides, particularly for miniaturized and integrated devices [7]. SPP is usually more easily excited in the surface of metal and graphene, which provide the desired plasmonic response at different frequency ranges. For metal materials, although the specific plasmon frequency is approximately linearly dependent on their geometric size [8], it is more general to synthetically tailor plasmon wavelengths of gold (Au) over the visible light spectrum [9, 10], while to adopt silver (Ag) and aluminum (Al) in the ultraviolet wavelengths [11, 12]. Intrinsic graphene shows interband absorption instead of plasmon modes excitation, due to the strong coupling association between quantum quenching and interband absorption in near-infrared and visible light regions [13]. Thus, the study of graphene plasmons mainly focuses on the mid-infrared to terahertz spectrum range [14–16]. For the near-infrared optical communication windows, particularly at 1.55 μm with the lowest transmission loss, the mentioned materials are no longer deemed suitable. Several studies have been reported to develop plasmonic waveguides with hybrid materials at 1.55 μm, based on traditional silicon-based optical waveguides. Guo et al. [17]. proposed a silicon–graphene hybrid plasmonic waveguide to achieve photodetector applications with tunable responsivity, which relies on the modulation effect of graphene plasmons. Ding et al. [18]. realized highly efficient electro–optic modulation in silicon-on-insulator platform waveguides modified by plasmonic Au and graphene. With the contribution of plasmons, a transmission loss optimization of 0.13 dB μm^−1^ (from 0.69 to 0.56 dB μm^−1^) was achieved at low gating voltages. However, due to the general mismatch of wave vector of existing plasmonic materials, the performance advantages of plasmonic waveguides at 1.55 μm near-infrared wavelength until now are still not attractive enough. It is of great significance to develop materials and waveguides with well-matched SPP properties in this band of concern.

Molybdenum (Mo) itself is a stable, high-melting point metal, simultaneously exhibiting excellent intrinsic physical properties, such as high electric and thermal conductivities [19, 20]. Self-assembled grown Mo nanostructures have been demonstrated to show significant light near-field localization and enhancement performance in a wide spectral range from visible to near-infrared [21–24]. More importantly, to excite SPP behaviour at the wavelength near 1.55 μm, Mo plasmonic materials theoretically do not require any form of pretreatments, such as voltage tuning or chemical doping [25]. Therefore, it is significant to fabricate a class of high-quality metallic Mo micro-nano structures and directly apply them to the study of optical waveguides by taking advantage of their SPP characteristics at 1.55 μm.

In this study, we successfully prepared perfect Mo microrod single-crystals, as the propagating main bodies of a high-performance near-infrared plasmonic waveguide, by means of a modified method of thermal evaporation physical vapor deposition. The excellent single-crystalline characteristics of the material guarantee the potential of SPP propagation. After optimized design and focused ion beam etching, we fabricated one-dimensional subwavelength grating structures on the surface of these Mo microrods, and then achieved attractive plasmonic waveguide performance exactly at 1.55 μm for the first time. The characterized propagation length of SPP is greater than 32 μm, which corresponds to a transmission loss of less than 0.067 dB μm^−1^. The results not only provide a novel plasmonic material for near-infrared waveguide and optical communication applications, but also expand people’s understanding of the optical properties and application design of transition metals.

## 2 Methods

### 2.1 Material preparation

Si, Al_2_O_3_, and high-purity Mo boat were cleaned with acetone and alcohol for more than 10 min. The high-purity Mo boat, as an evaporation source, was put inside a vacuum chamber, and the Al_2_O_3_ substrate was directly placed inside the boat. Initially, the base pressure of the vacuum chamber was evacuated to <5 Pa. High-purity argon (99.9 %, 200 sccm) and hydrogen (99.9 %, 100 sccm) were introduced into the vacuum chamber. After pressure stabilized, the temperature of the boat, controlled by a direct current flowing through it, was gradually increased to 1853 K with a rate of 150 K min^−1^. The temperature in the vacuum chamber was kept for more than 40 min. The hydrogen should be interrupted to guarantee the oxidation of molybdenum vapor, and the disproportionation of molybdenum dioxide proceeds smoothly [21–24]. After that, the temperature was decreased gradually to 300 K with holding hydrogen to prevent oxidation of the samples, and the Mo microrod single-crystals were synthesized.

### 2.2 Material characterization

The structure and intrinsic material properties of the samples were characterized by scanning electron microscope (SEM, Supra 60, Zeiss, operated at 10 kV), energy disperse spectroscope (EDS, X-Max, Oxford Instruments, operated at 20 kV), and transmission electron microscope (TEM, Titan, FEI, operated at 154 kV).

### 2.3 Simulations

FDTD simulations used commercial software (Lumerical FDTD Solutions). A TM-polarized plane wave was generally placed from the top of the subwavelength gratings. Detectors were placed upper and lower sides of the gratings to detect the reflection and transmission signal. Period boundary conditions (PBCs) were placed in both ±*y* and ±*z* directions, and perfectly matched layers (PMLs) were placed in ±*x* direction.

### 2.4 Optical property measurement

The near-field imaging was manifested with a scattering-type scanning near-field optical microscopy (s-SNOM, Neaspec GmbH, Quantum Scientific Instruments Trading Co., Ltd., Beijing). The near-field optical intensity distribution of Mo microrods could be mapped simultaneously with its surface morphology. The laser (1.55 μm) was illuminated onto the Mo microrod through a metal-coated tip (Arrow-IrPt, NanoWorld) to image the polariton waves. The scattered signal from the tip was detected by an MCT detector (HgCdTe, Kolmar Technologies).

## 3 Experimental results

**Figure 1:**
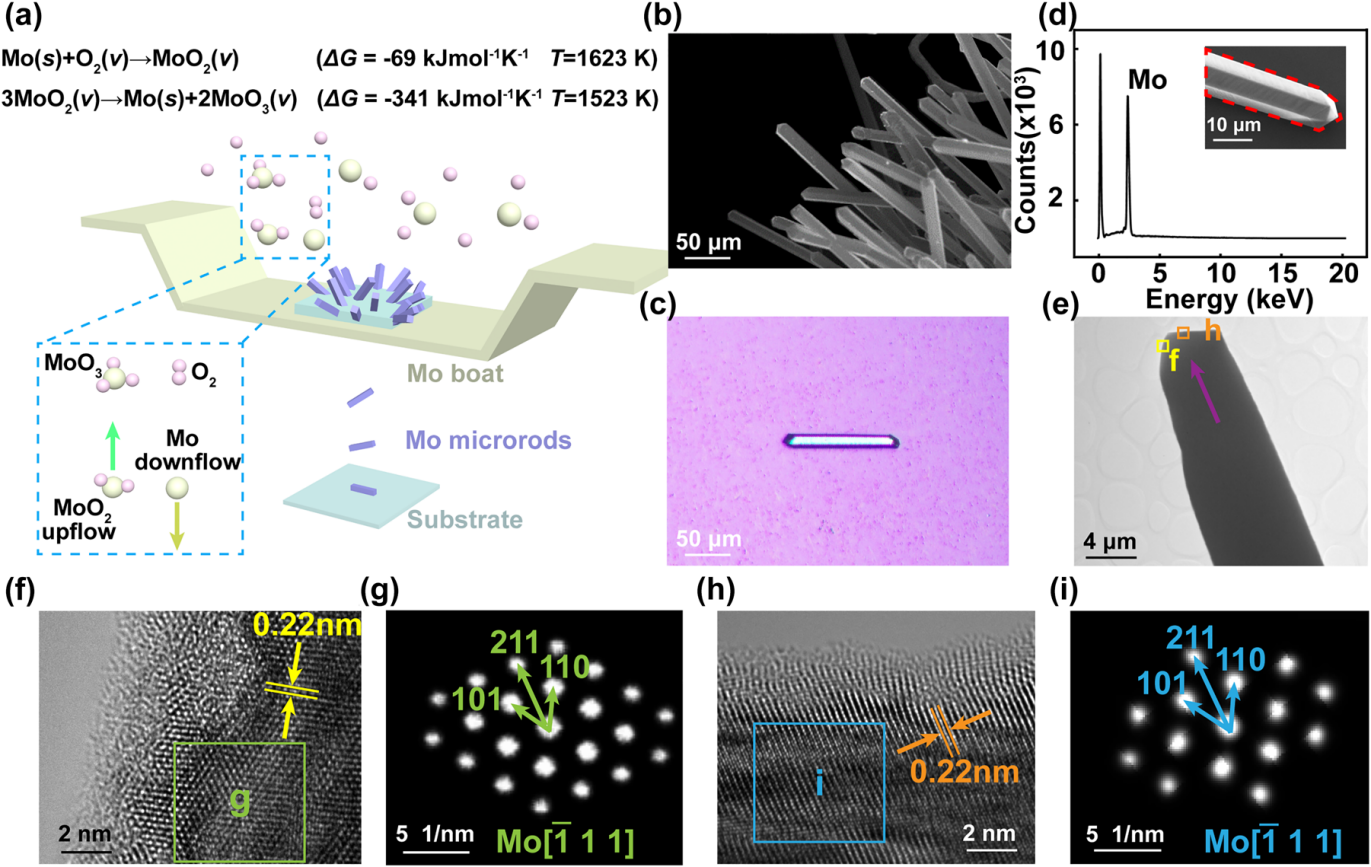
Characterizations of the prepared Mo microrod single-crystals. (a) Schematic diagram of the modified thermal evaporation physical vapor deposition to prepare Mo microrods. (b) SEM image of the Mo microrods. (c) Optical micrograph of an exfoliated Mo microrod. (d) EDS spectrum of the marked red area in the inset sample. (e) TEM image of a Mo microrod structure. The arrow indicates the direction of growth. (f) High-resolution TEM (HRTEM) image of area “f” in panel (e). (g) The corresponding FFT image of area “g” in panel (f). (h) HRTEM image of area “h” in panel (e). (i) The corresponding FFT image of area “i” in panel (h).

High-quality single-crystalline characteristics are one of the keys to ensuring the plasmonic propagation performance of materials [26, 27]. On the other hand, a quasi-one-dimensional rod shape is also conducive to the observation of SPP behavior in optical waveguide researches. Herein, the perfect Mo microrod single-crystals were successfully prepared through a series of reactions during a proposed modified physical vapor deposition. Two chemical reactions occurred throughout the above process, including the oxidation of Mo and the disproportionation decomposition of Mo dioxide (MoO_2_). The reaction equations are shown in Figure 1(a). Both chemical reactions could occur spontaneously, owing to the negative Gibbs free-energy values. At a temperature of 1623 K, gaseous MoO_2_ intermediates are generated by the Mo boat reacting with the residual oxygen. Then, Mo atoms decomposed from gaseous MoO_2_ are deposited on Al_2_O_3_ substrate. To obtain single-crystal products, the initial Mo atoms should follow the layered nucleation mode, with a growth rate stated as [28, 29]:

(1) $$R_{m-nuc}=aI_{0}^{1/3}\exp\left[-\frac{\pi {\left(\frac{\gamma^{\prime }}{kT}\right)}^{2}}{3\sigma }\right]v^{2/3}$$

where *a* denotes the step height of the Mo facets, *I*_0_ denotes the pre-exponential factor, *γ′* denotes the step energy per Mo adatom, *σ* denotes the degree of supersaturation, and *v* denotes the frequency factor. After initial nucleation, the growth rate of subsequent Mo rod along the side wall normal direction may be described as [30]:

(2) $$R_{\text{v}}=N_{0}v_{\text{n}}exp\left(-\frac{\Delta G_{\text{a}}}{kT}\right)\left\{\frac{1-exp\left[-R\ln\left(\sigma /k\right)\right]}{h}\right\}$$

where *N*_0_ is the number of growth positions available per unit area of the side wall of Mo rod, *v*_n_ is the frequency factor near the boundary between the side wall and the gas phase, *ΔG*_a_ is the barrier that the gas-phase element must cross to reach the structure, *R* is the gas constant, and *h* is the surface spacing of a crystalline plane on the side wall. In this step, the degree of supersaturation *σ* has a significant effect on the growth rate along both the orientations horizontal and vertical to the substrate, which will control the length and diameter of the Mo microrod single-crystals. Similar reaction mechanism and kinetic process have been discussed in detail aiming to another pyramidal Mo single-crystal in our previous work [22]. Specifically in this deposition preparation, the *σ* value of Mo can be controlled by adjusting the parameters such as evaporation source temperature and evaporation–condensation spacing to obtain the final rod-shaped structure. Figure 1(b) shows SEM image of the prepared Mo microrod structures. The average length of the microrod ranges from 100 to 200 μm, and some of them even show a maximum size of over 500 μm. These Mo microrods are distributed uniformly onto the surface of the Al_2_O_3_ substrate on account of lower Gibbs free energy here. These agglomerated Mo products were next exfoliated to a cleaned silicon dioxide (SiO_2_) wafer, obtaining separate microrods typically with length of over 100 μm (e.g. ∼107.2 μm) and diameter around 10 μm (e.g. ∼9.81 μm), as shown in Figure 1(c). Figure 1(d) shows energy disperse spectroscopy (EDS) of such an individual (marked in red in the inset), showing that the element percentage of Mo is 100 % after excluding the area of SiO_2_ substrate. Combined with the corresponding EDS-mapping results (Figure S1, Supporting Information), this proves that the generated microrod is composed of pure metallic Mo.

TEM characterization has been used to analyze the crystal structure and lattice arrangement of Mo microrods. Figure 1(e) shows a low-resolution TEM image, with a purple arrow revealing the growth direction of the microrod. The High-resolution TEM image of the yellow area marked “f” in Figure 1(e) proves that the Mo microrod is with good crystallization (Figure 1(f)). Similarly, another orange area marked “h” is shown in Figure 1(h), which reveals the lattice arrangement of the other side. Two adjacent planes indicate a mean lattice spacing of 0.22 nm, being consistent with the {110} planes of body-centered cubic (bcc) Mo structures. The fast Fourier transform images of marked areas “g” and “i” evidence a single-crystalline nature since these patterns are very ordered and sharp (Figure 1(g)–(i)). The nucleation direction can be determined along the [211] orientation. The excellent single-crystalline properties guarantee the potential of Mo microrod as an optical waveguide material. A plasmonic metal structure must be designed to support essential wave vector conservation to excite SPP onto the waveguide. Specifically, to excite a SPP on a metal surface, the wave vector component of the incident laser must equal the wavenumber of SPP. However, the laser that illuminates the metal surface could not directly couple to SPP. One can choose the following schemes to solve it. Firstly, the photon wave vector can be compensated in a total internal reflection geometry with a specific incidence angle [31]. Resonant tunneling occurs when photons couple to the metal surface polaritons. Notably, the excitation efficiency in such a scheme is sensitive to the thickness of the metal film, but the thickness of the prepared Mo rod structure herein is typically on micron scale and hard to excite an Otto-Kretschmann SPP behaviour. Secondly, SPP can also be launched on the metal surface through an illuminated tip, known as near-field excitation [32]. In this way, slight vibrations could cause the tip to collide with the metal surface, which might affect the stability of the system, not suitable for in-system waveguide applications. Thirdly, another way to excite SPP is to use a randomly rough surface, which presents diffracted components of all wave vectors in the near-field region [33]. However, the low coupling efficiency is a critical problem. The depth of the defect is also difficult to control during the thermal evaporation preparation in this study, which results in a complex electromagnetic field intensity distribution. Compared with all the above methods, a diffraction effect way is worth selecting due to its reliability and validity, to generate SPP for the prepared Mo microrod single-crystals [34–36]. An engraved periodic grating provides a well-defined spatial frequency component that mixes with the incident spatial frequency, so that there can be a strong component that matches the wavevector of the SPP, thus improving the generation efficiency. Diffraction on a grating provides the wave vector conservation as follows [34]:

(3) $$k_{\text{sp}}=\frac{\omega }{c}n_{\text{s}}sin\theta u_{12}\delta_{\text{p}}\pm p\frac{2\pi }{D}u_{1}\pm q\frac{2\pi }{D}u_{2}$$

where *n*_s_ is the refractive index of a metal. **u**_12_ is the unit vector in the direction of the incident light. *δ*_p_ depends on the incident light polarization angle. **u**_1_ and **u**_2_ are the unit lattice vectors of diffraction gratings, *D* is its period, and *p* and *q* are integer numbers. Therefore, the grating structure can be specifically designed to provide efficient coupling to air–metal SPP modes.

**Figure 2:**
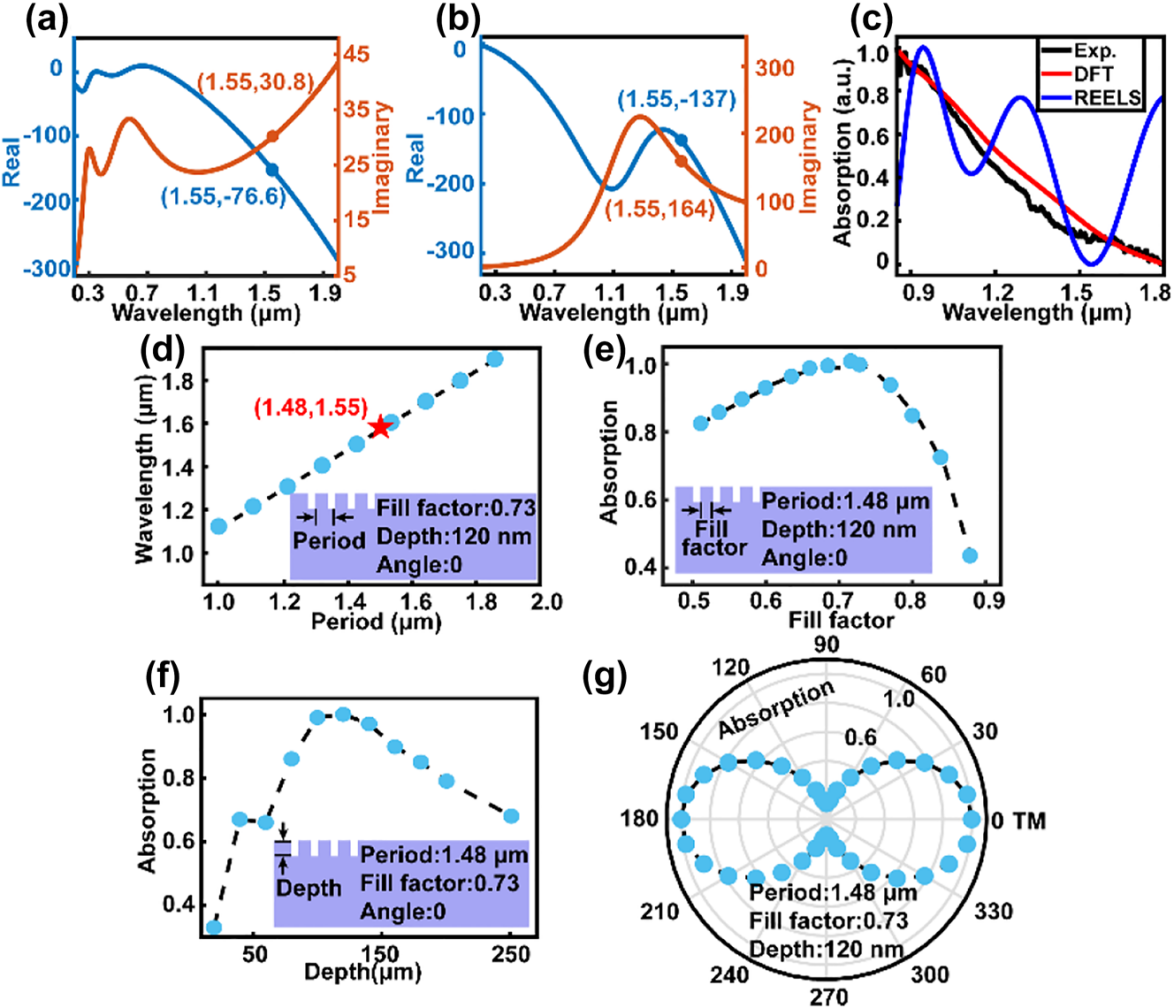
Mo plasmonic waveguide design and optimization. Permittivity of Mo obtained by (a) density functional theory (DFT) and (b) reflection electron energy loss spectroscopy (REELS). (c) Normalized absorption intensity versus wavelength curves for the experiment (black), DFT (red), and REELS (blue), respectively. (d) Calculated absorption characteristic wavelength as a function of the grating period. Calculated absorption intensity versus (e) the fill factor, (f) the grating depth, and (g) the polarization angle.

Design and optimization were thereafter employed with a finite-difference time-domain (FDTD) method to determine the Mo’s plasmonic waveguide structure. Before that, it is necessary to determine the permittivity of Mo. Figure 2(a) and (b) show two sets of optical values for the real and imaginary parts of the permittivity of Mo. One was calculated from the density functional theory (DFT) [25], and the other was obtained from the reflection electron energy-loss spectroscopy (REELS) [25]. Both of them gave optical constants from the near-ultraviolet to infrared range of wavelengths. To determine the permittivity of Mo, the DFT and REELS normalized absorption spectra based on the same geometric model were compared with the absorption spectrum experiments we conducted. The experimental data was identified by an UV-visible spectrometry (Shimadzu UV3600, Japan). As shown in Figure 2(c), the black, red, and blue represent the normalized absorption spectra of Mo microrods obtained from the experiment, DFT, and REELS, respectively. Since the DFT simulation model presented better validity and accuracy towards the experiment results, the DFT model was chosen for the next step of structural design and optimization.

Based on the Mo permittivity obtained from the DFT model, diffraction gratings onto the Mo microrod were then designed to match the external laser (∼1.55 μm) with the resonant wavelength approach to provide wave vector conservation. Figure 2(d) depicts the resonance wavelength as a function of the grating period (*λ*). The diffraction gratings were iteratively optimized and set with a fill factor (*f*) of 0.73, groove depth (*d*) of 120 nm, and polarization angle (*θ*) of 0. The trend of the curves varies linearly, and the resonance wavelength can be flexibly varied by modulating the grating period. A red feature point is highlighted in the Figure 2(d), which corresponds to the optimum *λ* of 1.48 μm for the 1.55 μm waveguide. In addition, the absorption spectrum in the grating structure is highly sensitive to changes in the geometrical parameters, such as groove depth, grating fill factor, and laser polarization angle. An analysis of the influence of these parameters aids structural optimization to achieve maximum coupling efficiency. Here, we first investigated the effect of grating fill factor (*f*) by varying it from 0.5 to 0.9 with fixing a period of 1.48 μm, groove depth of 120 nm, and polarization angle of 0, as shown in Figure 2(e). The simulation results show that the coupling efficiency increased with the grating fill factor and saturated at its maximum when the *f* is around 0.73. After that, the coupling efficiency decreased rapidly. Figure 2(f) shows the dependence of the coupling efficiency and the groove depth (*d*), with *λ* = 1.48 μm, *f* = 0.73, and *θ* = 0. The calculation result shows that the groove depth with *d* ≈ 100–140 nm could produce the highest efficiency. Thus, *d* around 120 nm was chosen as the groove depth of the gratings. The effect of external laser polarization angle was further studied. Within the context of this paper, we define the angle between the electrical field vector direction of the incident laser and the waveguide as the polarization angle. The electrical field vector direction is perpendicular to the grating lines. Figure 2(g) shows the absorption curves as a function of the polarization angle. The designed diffraction grating structure presents a distinct anisotropy of absorption. The calculated absorption value is maximum for TM polarized light and minimum for TE polarized light, which is consistent with the theoretical calculation results [37]. According to the simulation results, the diffraction grating parameters *λ* = 1.48 μm, *f* = 0.73, and *d* = 120 nm were finally chosen as the guideline for the fabrication of subwavelength diffraction gratings on the prepared Mo microrod single-crystals. External laser is selected TM polarization with a wavelength of 1.55 μm.

**Figure 3:**
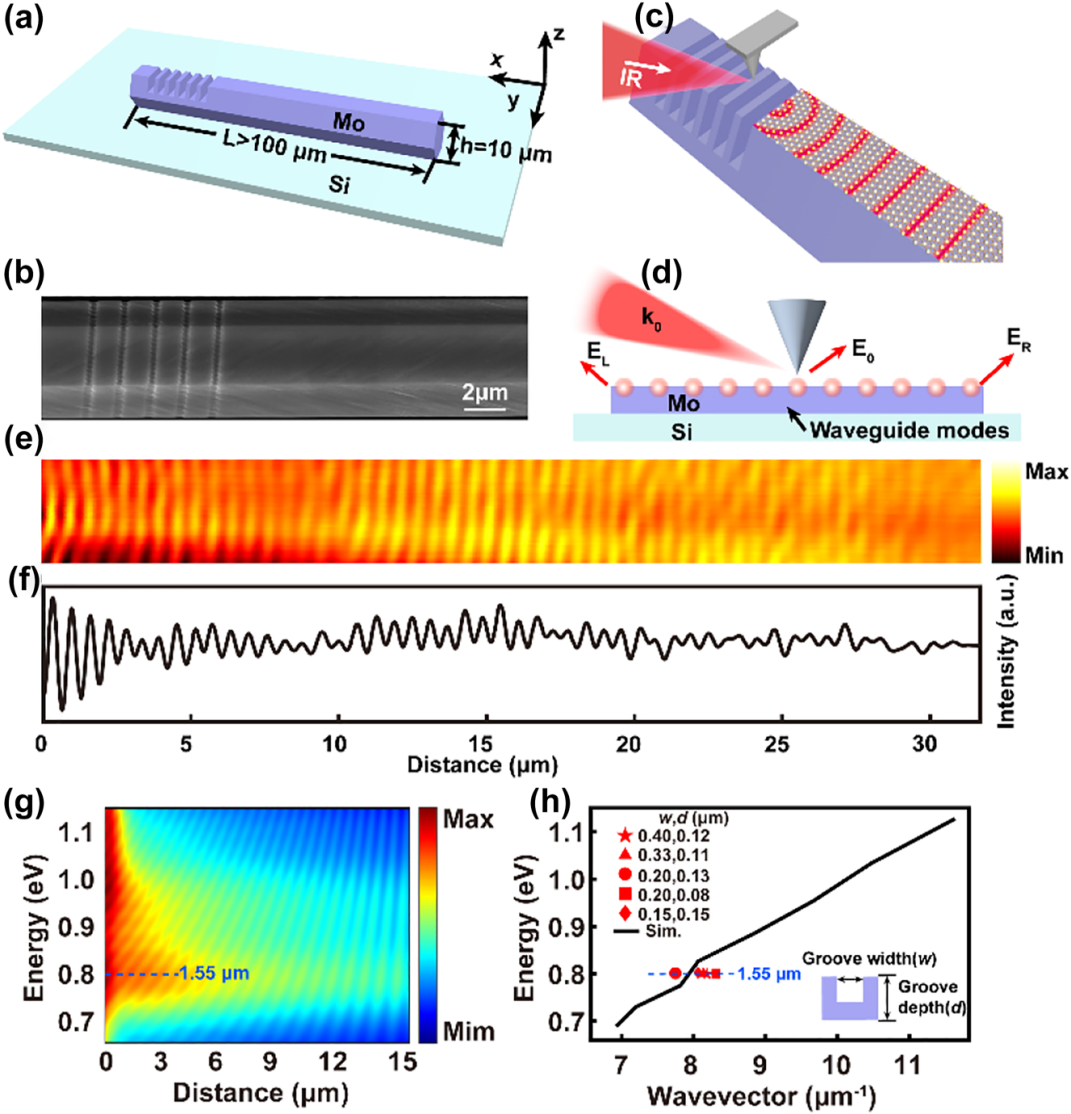
Real-space micro-imaging of the Mo plasmonic waveguide structure at 1.55 μm. (a) Schematic diagram of the Mo microrod waveguide with subwavelength gratings. (b) SEM image of the fabricated subwavelength gratings on the Mo microrod. (c) Schematic diagram of the SNOM experimental setup onto the Mo microrod. (d) Schematic diagram of the waveguide modes (red) on Mo microrod, in which *E*_0_ is the tip-scattered photons, and *E*_L_ and *E*_R_ are scattered photons after propagating to the edge in the microrod. (e) s-SNOM image of the Mo plasmonic waveguide with optimized subwavelength gratings. (f) Normalized near-field intensity profiles along the propagation direction. (g) Calculated SPP intensity distribution with various photon energy. (h) Dispersion relation obtained from the experiment (points) and simulation calculations (curve).

The Mo microrod plasmonic waveguide with artificially designed subwavelength gratings (shown in Figure 3(a)) has been demonstrated for the first time to show excellent low-loss surface plasmon propagation performance at 1.55 μm. Specifically, these diffraction gratings were successfully fabricated by a FIB/SEM (AURIGA) etching system onto a perfect Mo microrod single-crystal, which presented diameter around ∼10 μm and length of over 100 μm, and was located on a SiO_*2*_ (*x*–*y*) substrate. Locating the target Mo microrod in the SEM system, the edge of the Mo microrod was then set as the confocal point for FIB etching. The stage was tilted accordingly to ensure that the sample surface was always oriented perpendicularly to the ion beam during milling, with a working distance of 5 mm, accelerating voltage of 30 kV, and beam current of 2 pA. Figure 3(b) shows the SEM image of the fabricated gratings on the Mo single-crystal. AFM analysis shows that the etched gratings show *λ* of 1.43 μm, f of 0.748, and d of 120 nm, with an acceptable etching error (Figure S2, Supporting Information).

Waveguiding performances were evaluated to demonstrate the promising potential of the single-crystalline Mo microrod. The near-field optical technique could directly provide propagation of localized electromagnetic fields and optical information distribution within the waveguide. Figure 3(c) shows the schematic of waveguide modes (red) initiated by the s-SNOM tip. The lattice model ({110} facets) schematic, corresponding to the FFT images (Figure 1 g–i), is drawn on the surface of the Mo microrod. Photodetectors recorded the backscattered photons of the three primary pathways (*E*_0_, *E*_L_, and *E*_R_), as shown in Figure 3(d). The photons *E*_0_ are directly backscattered from the tip, which was detected in this measurement to analyze the near-field optical properties. The photons *E*_L_ and *E*_R_ propagate in the microrod surface and escape from the microrod edges, scattering into external space as far-field light. To improve the accuracy of the conclusion, the influence of near-field excitation and randomly rough surfaces must be excluded. The intrinsic Mo microrod without diffraction gratings was firstly characterized (Figure S3, Supporting Information). The bright areas in the detected SNOM image reveal that the electromagnetic field is significantly enhanced in these regions. However, the intrinsic Mo microrod with a rough surface under near-field excitation presented a complex electromagnetic field enhancement property that exhibiting diffracted components of all wave vectors.

Compared with the complex optical fields of the near-field excitation and rough surface excitation, distinct parallel streaks can be observed on the fabricated Mo plasmonic waveguide with diffraction gratings, as shown in Figure 3(e). These parallel streaks collected at the photodetector depend on the interference between the *E*_0_ and *E*_L_/*E*_R_. Additionally, due to the recombination loss during propagation in the Mo microrod single-crystal, it can be seen that the intensities of the streaks gradually decrease away from the diffraction grating. Here, the transmission loss of the Mo microrod can be calculated by ascertaining the propagation length, and the propagation modes of SPP can be further verified by the period of the fringes. Figure 3(f) shows the intensity profile corresponding to the near-field image along the propagation direction. The parallel fringes gradually disappear, and energy tends to be stabilized as the transmission distance increases, corresponding to the propagation length of 32 μm. The transmission loss (*α*) is calculated as 0.067 dB μm^−1^ by: $\alpha =-\frac{10}{Z\left[\mu m\right]}\log_{10}\frac{P\left(z\right)}{P\left(0\right)}$ , where *Z*[μm] is the propagation length, *P*(z) is the propagation intensity that decreases from the initial energy *P*(0) to e^−1/2^ times [38]. At other alternative unoptimized groove structures, the propagation loss rapidly increases by almost an order of magnitude (ranging from 0.241 to 0.542 dB μm^−1^) (Figure S4, Supporting Information). This suggest that the optimized groove structure can enhance the surface plasmon propagation on the single-crystalline Mo microrod plasmonic waveguide.

Figure 2(a) has shown the permittivity of Mo in the spectrum from 0.3 to 1.9 μm. One should know that when the wavelength is less than 0.8 μm, the SPP cannot be excited on the Mo structure because the real part of the permittivity is larger than the negative permittivity of air (*ε*_m_ > −*ε*_d_). While in the range of 0.8–1.0 μm, a higher imaginary part exists compared to the absolute value of the real part in the permittivity, indicating a large material loss. Therefore, the propagation characteristics of the SPP were calculated over the range of 0.65 eV (∼1.9 μm) to 1.15 eV (∼1.07 μm), as shown in Figure 3(g). The intensity distribution demonstrates that the optimized structure presents a relatively low transmission loss in the 1.55 μm. The dispersion relation, shown in Figure 3(h), was also calculated by extracting the fringe period of each spectrum in Figure 3(g). Several highlighted feature points are the experimental SPP wave vectors of different groove structures, all showing very close values (around ∼8 μm^−1^) to the theoretical calculation at 0.8 eV (corresponding to the 1.55 μm wavelength). This matching dispersion relationship demonstrates the low-loss SPP behaviour of Mo on its microrod waveguide structure with the optimized subwavelength gratings.

## 4 Conclusions

In summary, to develop a high-performance plasmonic waveguide for near-infrared wavelengths, we have successfully prepared Mo microrods with a high-quality single-crystalline structure by a modified thermal evaporation physical vapor deposition. These Mo microrod single-crystals were applied as the main body to excite SPP behaviour on their surface at 1.55 μm for the first time. The surface plasmon enhanced periodic propagation properties were further optimized by designing and etching with subwavelength diffraction gratings. The propagation distance was measured through a real-space micro-imaging technique to reach 32 μm with a low transmission loss of 0.067 dB μm^−1^. This attractive propagation behaviour suggests that Mo waveguides can be effectively excited and guide SPP in the near-infrared spectrum. Our approach opens a promising avenue to construct future photonic circuits and integrated optics devices.

## Supplementary Material

Supplementary Material Details

Supplementary Material Details

Supplementary Material Details
