# Supplementary material for: A low-loss molybdenum plasmonic waveguide: perfect single-crystal preparation and subwavelength grating optimization
Source: Nanophotonics. 2023 Oct 25;12(22):4185–93. doi: 10.1515/nanoph-2023-0480 (PMC11501364; doi:10.1515/nanoph-2023-0480)
Supplement: Supplementary file 2 — Supplementary Material Details [file j_nanoph-2023-0480_suppl_002.pdf]

Supplementary Materials

A Low-Loss Molybdenum Plasmonic Waveguide: Perfect Single-Crystal Preparation and Subwavelength Grating Optimization

Tao Cui, Yan Shen*, Ao Cheng, Runze Zhan, Zebo Zheng, Bo Tian, Jia Shi, Yanlin Ke, Lei Shao, Huanjun Chen, and Shaozhi Deng*

State Key Laboratory of Optoelectronic Materials and Technologies, Guangdong Province Key Laboratory of Display Material and Technology, School of Electronics and Information Technology, Sun Yat-sen University, Guangzhou 510275, China

*Corresponding Authors:
Yan Shen, e-mail: shenyan7@mail.sysu.edu.cn;
Shaozhi Deng, e-mail: stsdsz@mail.sysu.edu.cn

S1. EDS element mapping and corresponding atom percentages of Mo microrod single-crystal.

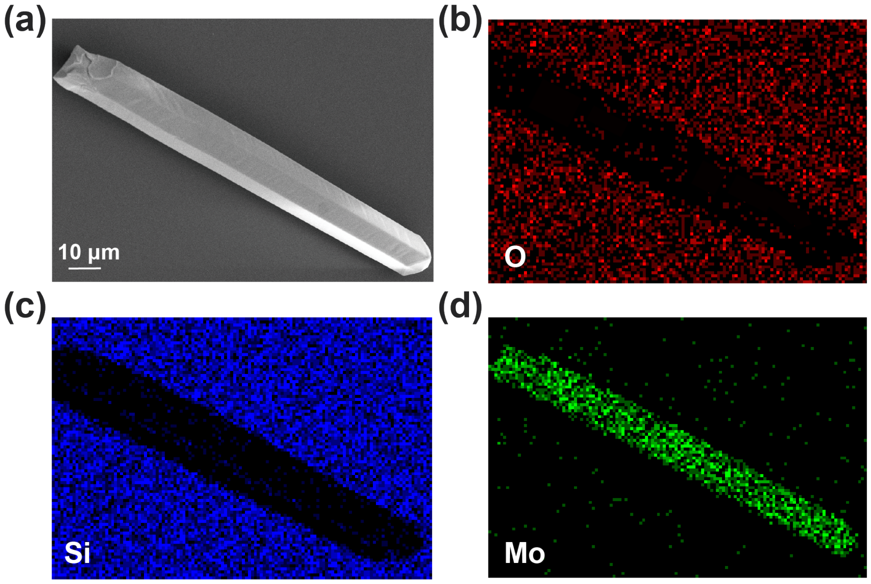

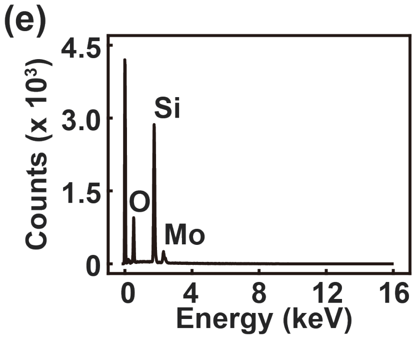

(f)

Element	Norm.wt.%	Norm at.%
O	31.69	49.95
Si	50.54	45.38
Mo	17.77	4.67
Sum	100.00	100.00

Figure S1. (a) SEM image of an exfoliated Mo microrod. (b), (c), and (d) The corresponding EDS mappings of O, Si and Mo. (e) EDS spectrum and (f) EDS quantification results of the prepared Mo microrod single-crystal.

S2. The grating structure characterization of Mo microrod after FIB etching.

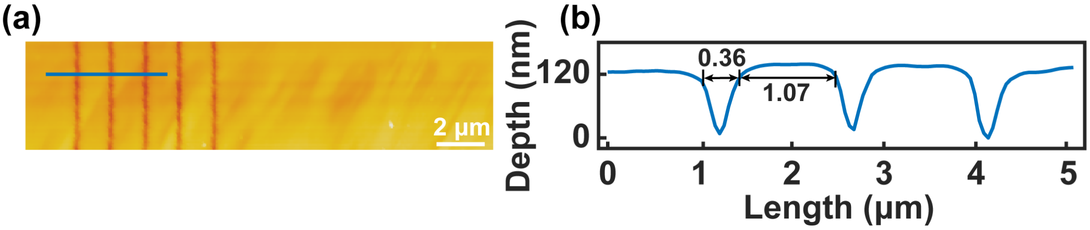

Figure S2. (a) AFM image of the fabricated subwavelength grating structure, (b) The grating structure characterization along the blue line in panel (a).

Figure S2 shows the AFM analysis of the optimized Mo microrod single-crystal to determine the grating period, fill factor and depth after FIB etching. The depth-sensing data along the blue curve was extracted and displayed in Figure S2(b). The results show that the fabricated grooves are with depth (d) of 120 nm, width of 360 nm, and period (λ) of 1.43 µm. The corresponding grating fill factor (f) was further calculated as 0.748, with an acceptable etching error to the as-designed structure.

S3. The near-field optical properties of the intrinsic Mo microrod single-crystal

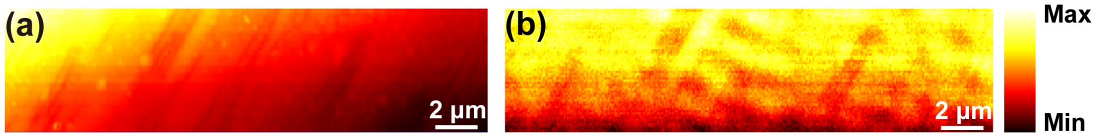

Figure S3. (a) AFM and (b) s-SNOM image of the intrinsic Mo microrod single-crystal.

Figure S3(a) depicts the AFM image of the intrinsic Mo microrod to characterize surface roughness. On a randomly rough surface, the excitation conditions for SPP can be achieved without any specific arrangements. Such SPP excitation processes result in a complex field distribution over the surface due to the interference between the SPP excited on rough interfaces of the film and the non-coupled illuminating light, as shown in the s-SNOM image of the intrinsic Mo microrod in Figure S3(b).

S4. Propagation length of the Mo plasmonic waveguides with different groove structures

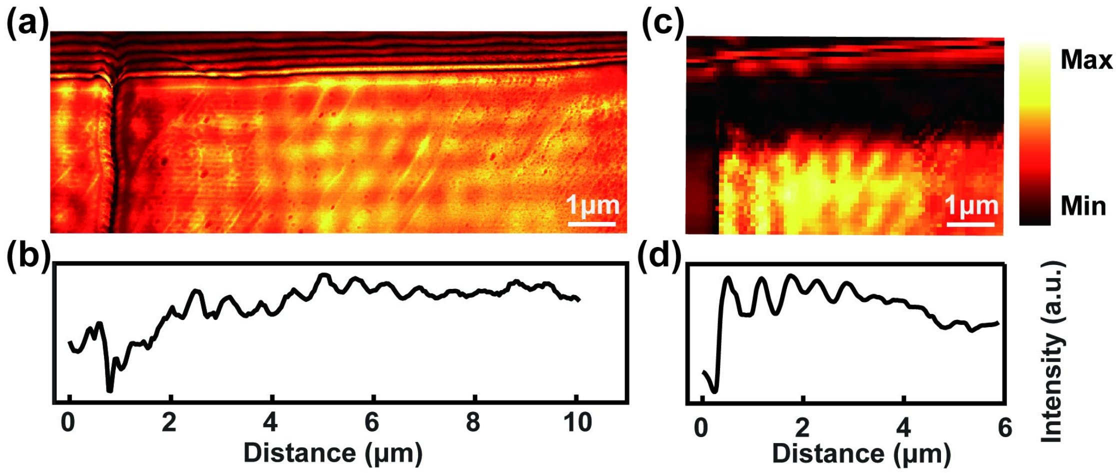

Figure S4. Representative s-SNOM images and near-field optical intensity distributions of different Mo microrod grating structures with (a) width of 330 nm, depth of 110 nm, and with (c) width of 200 nm, depth of 80 nm. (b), (d) Corresponding near-field intensity profiles along the propagation direction.

To demonstrate the effect of the different groove structures on the propagation length of the Mo microrod plasmonic waveguide, Figure S4 shows s-SNOM results of different Mo samples. From the near-field image of the 330 nm width and 110 nm depth Mo sample in Figure S4(a), one can see the intensity of the fringes gradually decreases when moving away from the groove edge for this sample. The intensity of parallel propagation is normalized in Figure S4(c), corresponding to a propagation distance of 9 μm. When the groove structure is etched to 200 nm width and 80 nm depth for another Mo sample, the propagation length will sharply decrease from 9 to 4 μm, corresponding to a loss increasing from 0.241 to 0.542 dB·μm^{-1}, as shown in Figs. S4 (b) and (d). The results suggest that the optimized groove structure can enhance propagation on a single-crystalline Mo microrod waveguide.
